# Metabolic alterations associated with cirrhosis after HCV cure in people with HIV: metabolomic and lipidomic findings at one and five years

**DOI:** 10.3389/fimmu.2026.1932664

**Published:** 2026-09-09

**Authors:** Ana Virseda-Berdices, Belén Fernández-Requena, Juan Berenguer, Juan Gónzalez-García, Carolina Gonzalez-Riano, Raquel Behar-Lagares, Cristina Díez, Victor Hontañón, Amanda Fernández-Rodríguez, Coral Barbas, Ruben Martin-Escolano, Salvador Resino, María Angeles Jiménez-Sousa

**Affiliations:** 1 Unidad de Infección Viral e Inmunidad, Centro Nacional de Microbiología, Instituto de Salud Carlos III, Majadahonda, Madrid, Spain; 2 Centro de Investigación Biomédica en Red en Enfermedades Infecciosas, Instituto de Salud Carlos III, Madrid, Spain; 3 Facultad de Medicina, Universidad Complutense de Madrid, Madrid, Spain; 4 Centro de Metabolómica y Bioanálisis, Facultad de Farmacia, Universidad San Pablo-CEU, CEU Universities, Urbanización Montepríncipe, Boadilla del Monte, Spain; 5 Unidad de Enfermedades Infecciosas/VIH, Hospital General Universitario Gregorio Marañón, Madrid, Spain; 6 Instituto de Investigación Sanitaria Gregorio Marañón, Madrid, Spain; 7 Servicio de Medicina Interna–Unidad de VIH, Hospital Universitario La Paz, Madrid, Spain; 8 Instituto de Investigación Sanitaria La Paz, Madrid, Spain

**Keywords:** chronic hepatitis C, HCV therapy, HIV, liver cirrhosis, metabolomics, sustained virologic response

## Abstract

**Background & aims:**

People with HIV (PWH) who achieve hepatitis C virus (HCV) cure may retain persistent metabolic alterations, particularly those with advanced fibrosis or cirrhosis. This study aimed to characterize plasma metabolomic and lipidomic profiles associated with cirrhosis in PWH at one and five years post-HCV therapy.

**Methods:**

Two cross-sectional studies evaluated PWH one (n=48) and five (n=30) years post-HCV therapy. Cirrhosis was defined as a liver stiffness measurement (LSM)≥12.5 kPa. Metabolomics and lipidomics were performed using capillary electrophoresis-mass spectrometry (CE-MS) and liquid chromatography-mass spectrometry (LC-MS), respectively. Data were analyzed using orthogonal partial least squares discriminant analysis (OPLS-DA) and generalized linear models (GLM), adjusting for relevant covariates.

**Results:**

At one and five years, 32 (66.7%) and 10 (33.3%) participants, respectively, had cirrhosis. OPLS-DA identified 235 and 229 metabolites with variable importance in projection (VIP) scores >1. At one year, cirrhosis was associated with elevated levels of glycerophospholipids, sphingomyelins, and amino acids, and lower levels of triglycerides. At five years, cirrhotic PWH exhibited higher levels of glycerophospholipids and acyl-carnitines, together with lower levels of triglycerides and amino acids.

**Conclusions:**

PWH with cirrhosis post-HCV cure exhibits a persistently altered metabolic profile stable for five years, suggesting ongoing systemic inflammation and liver disease progression. These findings underscore the need for continued long-term monitoring of this population.

## Introduction

Sustained virologic response (SVR), whether achieved through interferon (IFN)-based regimens or all-oral direct-acting antivirals (DAAs), profoundly alters the natural history of hepatitis C virus (HCV) infection (1). SVR reduces the risk of progression to cirrhosis, hepatic decompensation, hepatocellular carcinoma (HCC), and both liver-related and all-cause mortality (2, 3). Beyond hepatic outcomes, SVR is also associated with a lower incidence of extrahepatic manifestations, including cardiovascular events as well as renal and metabolic complications (2, 3).

Nevertheless, a subset of patients with advanced fibrosis or cirrhosis continue to experience disease progression and remain at residual risk of HCC (4). This risk is particularly pronounced in the presence of additional liver-damaging factors, such as excessive alcohol consumption (5), metabolic dysfunction–associated steatotic liver disease (MASLD) (6), or coinfection with hepatotropic viruses, including hepatitis B virus (HBV) with or without hepatitis D virus (HDV) (7). These findings highlight the importance of continued surveillance in patients with advanced liver disease, even after HCV clearance (8).

Recent advances in metabolomics and lipidomics offer powerful tools to unravel the molecular mechanisms underlying liver disease pathophysiology. Comprehensive profiling of metabolites and lipids can reveal perturbations in pathways related to inflammation, fibrogenesis, and hepatic dysfunction, and may enable the identification of biomarkers predictive of disease progression and clinical outcomes (9, 10). These metabolic disorders have been found in several viral infections (11), including HIV. People with HIV (PWH) represent a particularly vulnerable population. HIV infection disrupts multiple metabolic pathways, and although antiretroviral therapy (ART) partially restores them, persistent alterations and chronic immune activation may contribute to long-term hepatic and metabolic complications (12, 13).

This study aims to investigate the metabolomic and lipidomic profiles of PWH who continue to present with cirrhosis despite achieving HCV cure. By characterizing molecular alterations in this high-risk population, we aim to shed light on the residual mechanisms driving liver disease progression after viral eradication.

## Methods

### Study subjects

We conducted two cross-sectional studies including PWH on long-term suppressive ART: 48 participants assessed one year, and 30 participants assessed five years after successful completion of HCV therapy, with 24 individuals assessed at both time points. HCV treatment regimens included either IFN-free DAAs or IFN-based therapy (peg-IFN-α/ribavirin or peg-IFN-α/ribavirin/DAAs). Participants were recruited from 10 centers across Spain between 2012 and 2021 (Marathon Study Group; Appendix A).

SVR was defined as an undetectable HCV-RNA load 12–24 weeks after treatment completion. All patients were receiving stable ART for at least six months and had an undetectable plasma HIV viral load (<50 copies/mL). Individuals with acute hepatitis C, hepatitis B virus (HBV) infection, or a prior history of liver decompensation were excluded.

This study was approved by the Research Ethics Committee of the Institute of Health Carlos III (CEI PI 72_2021) and conducted in accordance with the Declaration of Helsinki. All participants gave their written informed consent before enrollment.

### Clinical data and samples

Epidemiological and clinical data were retrospectively collected using a secure online platform, and subsequently verified against patient medical records to ensure accuracy and consistency. LSM were performed by trained operators using transient elastography, as previously described (14).

Peripheral blood samples were collected in ethylenediaminetetraacetic acid (EDTA) tubes, and plasma was isolated by Ficoll-Paque density gradient centrifugation. Samples were stored at -80°C in the Spanish HIV HGM Biobank until shipment for analysis.

### Primary study variable

The primary study variable was the presence of cirrhosis, defined as LSM≥12.5 kPa. This threshold was selected because it is a widely validated and commonly used cutoff for the non-invasive diagnosis of cirrhosis (15). Significantly, transient elastography is validated in viral hepatitis and shows equivalent performance in hepatitis C, hepatitis B, and HIV/HCV coinfection, according to the EASL-ALEH Clinical Practice Guidelines on non-invasive assessment of liver disease (16). Data were analyzed separately for the cross-sectional assessment performed at one year and five years after completion of HCV therapy.

### Non-targeted metabolomics analysis

A detailed description of metabolite extraction, reagents and standards, quality control (QC), procedures, analytical conditions, and metabolite annotation is provided in **Supplementary Data 1**.

### Metabolites extraction and quality assurance

Inactivated plasma samples were prepared at the Centro de Metabolómica y Bioanálisis, CEMBIO (Madrid, Spain). For CE-MS analysis, the procedure involved drying on the SpeedVac Concentrator (Thermo Fisher Scientific, Waltham, MA, USA), a deproteinization process, and transference to a chromacol vial. For LC-MS, processed plasma samples were extracted using a procedure that included methanol and methyl-tert-butyl ether (MeOH: MTBE (1:1, v/v)) (17).

For quality management assurance and blank samples, each plasma sample was polled in equal quantities to create QC samples. These QC samples were processed in the same way as the other samples. To detect common contaminations, two blank samples were prepared and processed using the same lipid extraction method as the other samples. The analytical sequence started and ended with injections of these samples.

### Capillary electrophoresis analysis (CE-MS)

We carried out an untargeted metabolomics-based approach to capture the diverse spectrum of plasma metabolites. Agilent CE 7100 coupled with an Agilent 6224 time-of-flight Mass Spectrometer (TOF-MS) analyzer was used to analyze the samples. We used a fused-silica capillary (Agilent Technologies; total length 100 cm x 50 μm i.d. x 360 µm) for CE-MS separation analysis in positive ionization mode. ESI (+) positive polarity mode was used in mass spectrometer (MS). Finally, CE-MS system was operated using MassHunter Workstation 6200 series TOF version B.09.00 c(B9044.0) acquisition software.

### Lipidomics analysis (LC-MS)

Agilent 1290 Infinity II Ultra-High-Performance Liquid-Chromatography (UHPLC) system coupled to an Agilent 6546 Quadrupole Time-of-Flight (QTOF) Mass Spectrometer (MS) equipped with dual Agilent Jet Stream (AJS) Electrospray (ESI) ion source (separate runs in positive and negative ESI modes) was used to analyze the samples. We used MassHunter Workstation Software LC-MS Data Acquisition v B.09.00 (Agilent Technologies, Waldrobonn, Germany) to obtain MS/MS information for plasma lipidome.

### Data reprocessing

Hybrid strategies were followed for the reprocessing of the raw data files, consisted of using an extensive compound database, both lipidomics and metabolomics data, respectively, which was imported into the Agilent MassHunter Profinder software (B.10.0.2, Agilent Technologies, Santa Clara, CA, USA) to perform the time alignment and feature extraction using the “Batch Targeted Feature Extraction” mode. For CE-MS data reprocessing we employed an in-house library (18), included in the CEU Mass Mediator (CMM) online tool (19).

To construct the lipidomic database, we performed a comprehensive lipid annotation process using four bioinformatic tools. This process incorporates a bottom-up strategy (LipidHunter), spectra matching (Lipid Annotator, MS-Dial), and the fragment intensity rules (LipidMS) (20). Then, false software annotations and redundant data were removed following the review of tentative annotations supplied by the software tools. To obtain a final list of lipids, we used the assistance of the CEU Mass Mediator (CMM) (19) and the manual inspection of the MS and MS/MS spectra data.

**Supplementary Data 1** shows full description of the software parameters. The integration of multiple complementary tools enhanced the accuracy and reliability of the lipid annotation.

Databases included the migration time (MT), monoisotopic exact mass, and molecular formula. Only features that were present in at least 50% of the samples within a single sample group, were taken into consideration as a last post-processing filter.

For CE-MS metabolomics data, the methionine sulfone IS, which was added in the initial preparation phase, was used for normalization. Lipidomic data normalization was carried out using the Kuligowski transformation (21). Then, we applied a threshold of 30%, based on the coefficient of variation (CV) within the QC samples, to select metabolites and lipid species.

### Statistical analysis

Statistical analysis was performed using MetaboAnalyst 6.0 software (http://www.metaboanalyst.ca/) and R software version 4.3.1 (R Foundation for Statistical Computing, Vienna, Austria).

For descriptive analysis, categorical variables were expressed as absolute counts (percentages), and continuous variables were shown as median and interquartile range (IQR). Chi-square and Mann-Whitney U tests were used to compare categorical and quantitative variables, respectively.

For the multivariate analysis, firstly, metabolomic variables were log-transformed (log_2_) and auto-scaled. Next, we performed a supervised multivariate analysis by orthogonal partial least squares discriminant analysis (OPLS-DA), which models simultaneously all features. Variable importance in projection (VIP) scores were calculated for each feature and model validity was assessed using a 1,000-permutation test.

For the association analysis, Generalized Linear Models (GLM) with gamma distribution was performed to evaluate the association between individual metabolomic features (dependent variable) and cirrhosis (LSM≥12.5 KPa) (independent variable) one year and five years after successful completion of HCV therapy. The models were adjusted for relevant covariates (age, gender, BMI, and HCV treatment) using a forward stepwise selection method based on the lowest Akaike Information Criterion (AIC). Results were reported as the adjusted arithmetic means ratio (aAMR) and corresponding p-values. Multiple testing correction was performed using the false discovery rate (FDR) according to Benjamini and Hochberg procedure. Statistical significance was defined as p-value <0.05 and an FDR-adjusted p-value (q-value) <0.2. With significant metabolites present at both time points, we performed a longitudinal within-individual analysis in the subset of patients with paired samples at both time points (n=24). Generalized Linear Mixed Models (GLMM) with a gamma distribution and log-link function were applied. We assessed the temporal evolution of metabolites in relation to liver stiffness, including visit (year 1 vs. year 5), continuous LSM value, and their interaction term (visit * LSM) as fixed effects. The coefficient of this interaction term (expressed as AMR) was used to determine whether the association between significant metabolites and LSM increased or attenuated from year one to year five in the same individuals.

### Lipid pathways

Functional associations of metabolites significatively associated to cirrhosis were analyzed and visualized by Lipid Network Explorer platform (LINEX) (22). The logarithm of fold change (LFC) was used to quantify differences in lipid abundances and to visualize variation patterns across groups.

## Results

### Patient characteristics

Clinical and epidemiological characteristics of the 48 PWH assessed one year after completion HCV treatment and of the 30 individuals evaluated five years post-treatment are summarized **Table 1**.

**Table 1:** Epidemiological and clinical characteristics of people with HIV (PWH) cured of HCV, stratified by cirrhosis status one and five years post-HCV therapy.

	One-year post-HCV therapy			Five-years post-HCV therapy		
	Without cirrhosis	With cirrhosis	p-value	Without cirrhosis	With cirrhosis	p-value
**No.**	16/48	32/48		20/30	10/30	
Age (years)	54 (52-56)	52 (50-55)	0.470	57 (53-60)	56 (53-58)	0.559
Gender (male)	13(81.2%)	25 (78.1%)	0.999	17 (85.0%)	6 (60.0%)	0.285
BMI (kg/m^2^)	24.0 (21.4-25.9)	24.9 (21.7-27.6)	0.481	24.5 (21.5-27.1)	22.8 (21.9-25.7)	0.502
Smoker (n=45/n=29)			0.678			0.702
Never	2 (12.5%)	3 (10.3%)		1 (5.0%)	1 (11.1%)	
Previous (>6 months)	4 (25%)	11 (37.9%)		7 (35.0%)	2 (22.2%)	
Current	10 (62.5%)	15 (51.7)		12 (60.0%)	6 (66.7%)	
Alcohol intake (>50g/day) (n=46/n=27)			0.238			0.601
Never	11 (68.8%)	15 (50%)		10 (55.6%)	4 (44.4%)	
Previous (>6 months)	5 (31.2%)	11 (36.7%)		7 (38.9%)	5 (55.6%)	
Current	–	4 (13.3%)		1 (5.6%)	–	
Intravenous drug user (n=46/n=26)			0.378			0.966
Never	6 (40%)	7 (22.6%)		3 (18.8%)	1 (10.0%)	
Previous (>6 months)	9 (60%)	24 (77.4%)		13 (81.2%)	9 (90.0%)	
Current						
Liver markers						
LSM (kPa)	7.1 (6.0-8.3)	18.6 (14.3-27.9)	**<0.001**	7.7 (6.0-9.2)	18.6 (15.4-26.9)	**<0.001**
HSI (n=37/n=27)	31.2 (30.1-33.8)	32.1 (28.5-36.9)	0.947	33.5 (28.7-39.4)	31.6 (28.0-33.9)	0.283
HCV markers						
HCV genotype (n=47/n=30)			0.282			0.502
1	12 (75%)	22 (71.0%)		16 (80.0%)	6 (60.0%)	
3	4 (25%)	4 (12.9%)		2 (10.0%)	3 (30.0%)	
4	–	5 (16.1%)		2 (10.0%)	1 (10.0%)	
HCV therapy			<0.001			0.628
pegIFN	15 (93.8%)	13 (40.6%)		17 (85.0%)	7 (70.0%)	
DAAs	1 (6.2%)	19 (59.4%)		3 (15.0%)	3 (30.0%)	
HIV markers						
Previous AIDS (n=38/n=30)	1 (6.2%)	0	0.734	1 (5.0%)	1 (10.0%)	0.999
CD4+ T-cells/mm^3^	536 (348-710)	487 (273-714)	0.705	481 (329-620)	372 (277-670)	0.846
CD4+ T-cells < 500 cells/mm^3^	6 (37.5%)	17 (53.1%)	0.475	10 (50.0%)	6 (60.0%)	0.897
HIV antiretroviral therapy			0.491			0.362
NRTI + NNRTI	6 (37.5%)	11 (34.4%)		7 (35.0%)	3 (30.0%)	
NRTI + II	4 (25.0%)	14 (43.7%)		5 (25.0%)	1 (10.0%)	
NRTI + PI	4 (25.0%)	3 (9.4%)		4 (20.0%)	5 (50.0%)	
PI+II+NNRTI/MVC	–	1 (3.1%)		–	–	
Others	2 (12.5%)	3 (9.4%)		4 (20.0%)	1 (10.0%)	

Data are presented as n (%) for categorical variables and median (interquartile range, IQR) for continuous variables. HIV-related markers (Previous AIDS, CD4+ T-cells, and HIV antiretroviral therapy) reflect the patient’s status prior to the initiation of HCV therapy. For variables with incomplete data, the number of individuals with available data at each time point is shown in parentheses (one year/five years after HCV therapy).

AIDS, acquired immune deficiency syndrome; BMI, body mass index; DAAs, direct-acting antivirals; HCV, hepatitis C virus; HIV, human immunodeficiency virus; HSI, hepatic steatosis index; II, HIV integrase inhibitor; IQR, interquartile range; kPa, kilopascal; LSM, liver stiffness measurement; NNRTI, non-nucleoside reverse transcriptase inhibitor; NRTI, nucleoside reverse transcriptase inhibitor; pegIFN, pegylated interferon; PI, HIV protease inhibitor.

Significant differences between groups appear in bold. Values in bold indicate statistically significant differences between groups (p-value <0.05).

At one-year time point, 32 individuals (67%) had cirrhosis. Significant differences between patients with and without cirrhosis were observed in LSM and type of HCV treatment (p<0.001 for both). At the five-year assessment, 10 individuals (33%) had cirrhosis, with significant differences between groups in both LSM and FIB-4 score (p<0.001 for both).

The evolution of LSM from baseline to one- and five-years is displayed in **Supplementary Data 2**. In addition, significant differences were found in platelet counts, international normalized ratio (INR), and aspartate aminotransferase (AST) levels at one year, as well as in hemoglobin levels at five years after HCV therapy (**Supplementary Data 3**, **4**).

### Metabolite detection in plasma samples

A total of 646 plasma metabolites were detected across the different platforms: 80 by CE-MS, 440 by LC-MS ESI+ and 126 by LC-MS ESI-. In the CE-MS analysis, most identified metabolites were amino acids (33.8%) and amino acid derivatives (45.0%). Based on the LIPID MAPS Structure Database (LMSD), lipid species were classified into four main categories, as detailed in **Supplementary Data 5**. In total, 112 glycerolipids (GL) (19.8%), 286 glycerolphospolipids (GP) (50.6%), 92 sphingolipids (SP) (16.3%), and 74 fatty acyls (13.1%) were identified.

### Metabolic profiling associated with cirrhosis one year after post-HCV therapy

**Figure 1:**
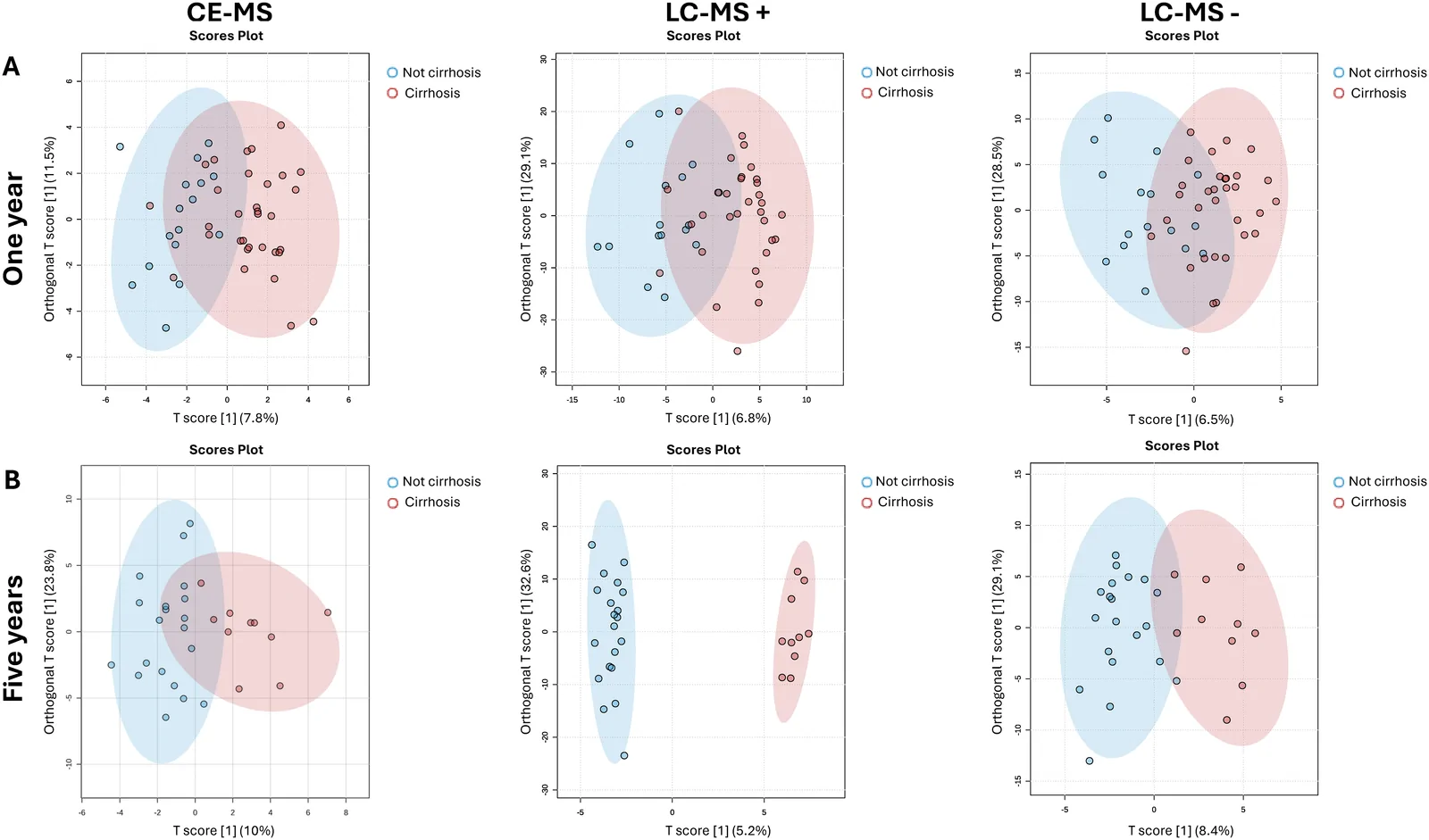
Orthogonal partial least square discriminant analysis scores plots discriminating between PWH with and without cirrhosis. The plots show results from three different analytical platforms: (left) Capillary Electrophoresis-Mass Spectrometry (CE-MS), (center) Liquid Chromatography-Mass Spectrometry in positive ion mode (LC-MS+), and (right) LC-MS in negative ion mode (LC-MS-). Panels show analysis at **(A)** one year and **(B)** five years post-HCV therapy. DG, diglyceride; FA, fatty acid; FAHFA, fatty acyl ester of hydroxy fatty acid; LPC, lysophosphatidylcholine; LPE, lysophosphatidylethanolamine; MG, monoglyceride; PA, phosphatidic acid; PC, phosphatidylcholine; PE, phosphatidylethanolamine; PI, phosphatidylinositol; PS, phosphatidylserine; SM, sphingomyelin; TG, triglyceride.

**Figure 2:**
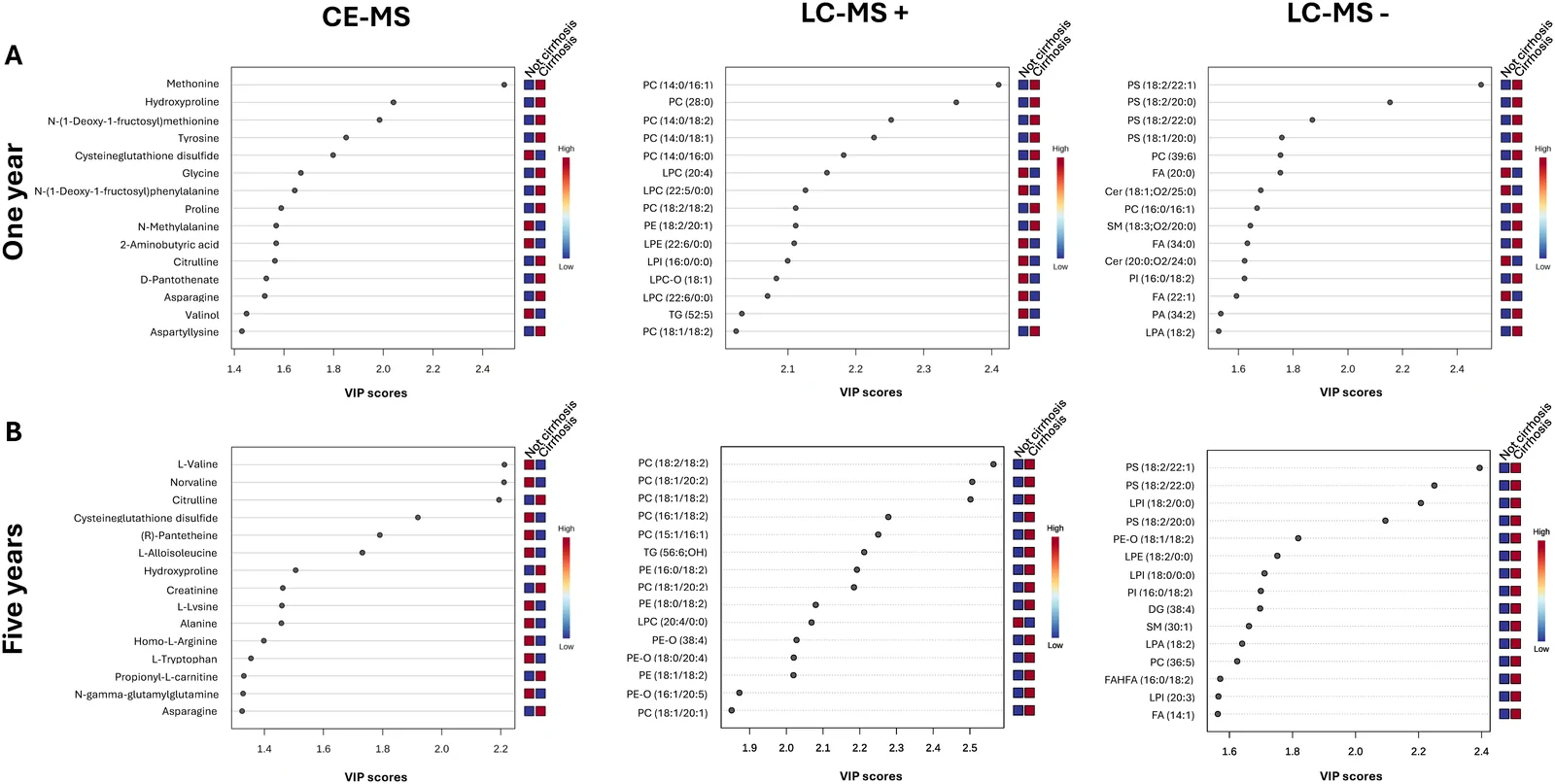
Top 15 metabolites with the highest variable’s importance (VIP) scores (≥1) from OPLSDA models. The plots show the most influential features discriminating between PWH with and without cirrhosis at **(A)** one year and **(B)** five years post-HCV therapy, separated by analytical platform. The color scale indicates the relative abundance of each metabolite: red bars indicate higher abundance, and blue bars indicate lower abundance. DG, diglyceride; FA, fatty acid; FAHFA, fatty acyl ester of hydroxy fatty acid; LPC, lysophosphatidylcholine; LPE, lysophosphatidylethanolamine; MG, monoglyceride; PA, phosphatidic acid; PC, phosphatidylcholine; PE, phosphatidylethanolamine; PI, phosphatidylinositol; PS, phosphatidylserine; SM, sphingomyelin; TG, triglyceride.

**Figure 3:**
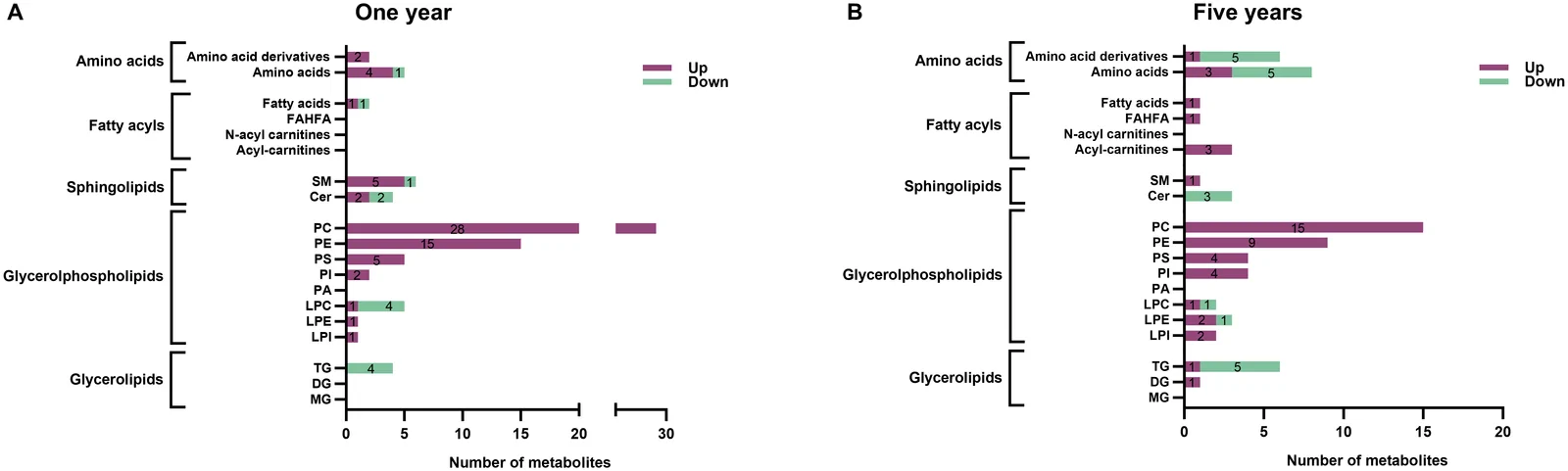
Significantly altered metabolites in individuals with cirrhosis. The bar charts display the number of metabolites that were significantly increased (purple) or decreased (green) in PWH with high versus low LSM at **(A)** one year and **(B)** five years post-HCV therapy. Metabolites are grouped by class. Statistical analysis: Significance was determined using Generalized Linear Models (GLM), adjusted for age, gender, BMI, and HCV treatment. Cer, ceramides; DG, diglyceride; FAHFA, fatty acyl ester of hydroxy fatty acid; LPC, lysophosphatidylcholine; LPE, lysophosphatidylethanolamine; LPI, lysophosphatidylinositol; MG, monoglyceride; PC, phosphatidylcholine; PE, phosphatidylethanolamine; PI, phosphatidylinositol; PS, phosphatidylserine; SM, sphingomyelin; TG, triglyceride.

OPLS-DA models effectively discriminated patients according to cirrhosis status, identifying 235 plasma metabolites with VIP scores >1 (**Figures 1A**, **2A**). Subsequent GLM analysis identified 80 metabolomic features significantly associated with cirrhosis. Among these, 13 features were found at lower proportion (AMR <1), and 67 at higher proportion (AMR ≥1) in PWH with cirrhosis. As shown in **Figure 3A**, significant metabolites detected by CE-MS included five amino acids and two derivatives. Lipids, identified by LC-MS were distributed across four categories: fatty acyls (2 compounds), SP (10), GP (54), and GL (4). Detailed association data for significant metabolites and lipids are shown in **Supplementary Data 6**.

**Figure 4:**
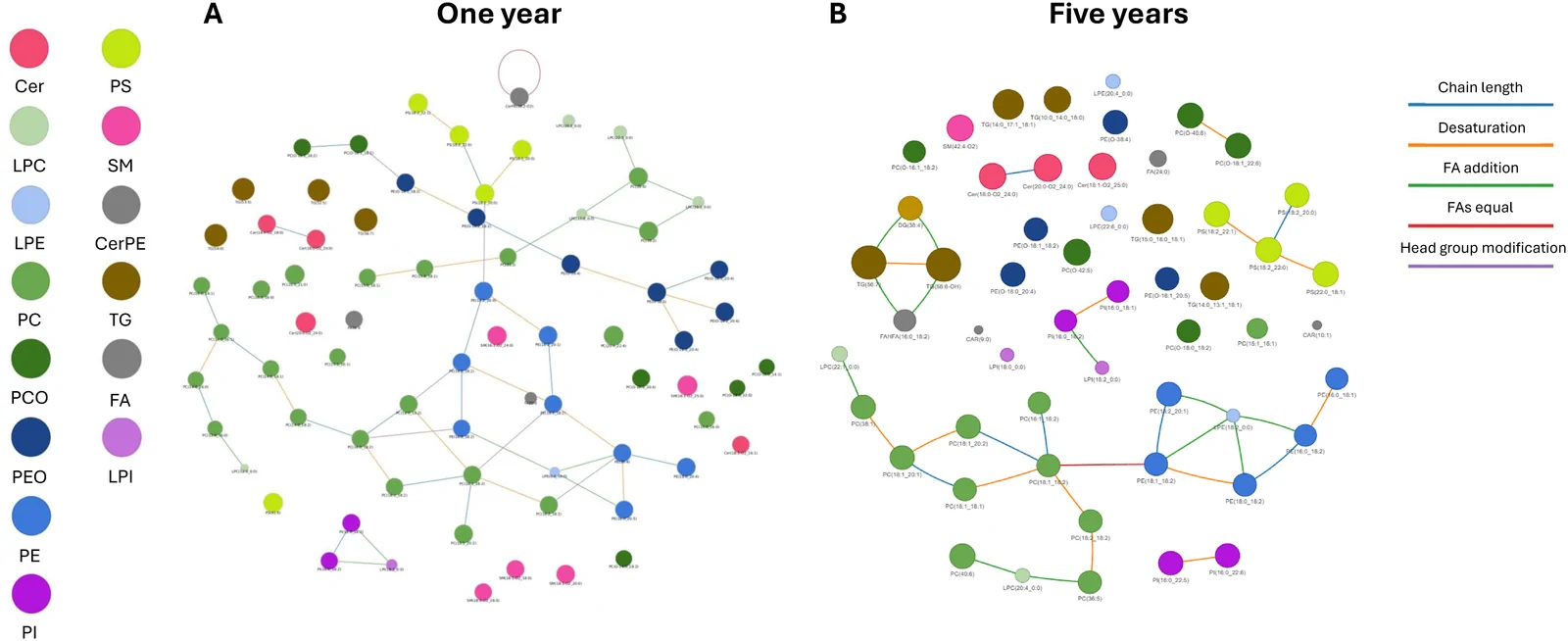
Lipid network analysis of metabolites associated with cirrhosis. The networks illustrate the biochemical relationships between significantly altered lipids in PWH at **(A)** one year and **(B)** five years post-HCV therapy. Nodes represent individual lipid species (colored by lipid class), and edges represent biochemical reactions connecting them (colored by reaction type). Cer, ceramide; CerPE, ceramide-phosphate-ethanolamine; DG, diglyceride; FA, fatty acid; FAHFA, fatty acyl ester of hydroxy fatty acid; LPC, lysophosphatidylcholine; LPE, lysophosphatidylethanolamine; LPI, lysophosphatidylinositol; MG, monoglyceride; PA, phosphatidic acid; PC, phosphatidylcholine; PCO, ether-linked phosphatidylcholine; PE, phosphatidylethanolamine; PEO, ether-linked phosphatidylethanolamine; PI, phosphatidylinositol; PS, phosphatidylserine; SM, sphingomyelin; TG, triglyceride.

Higher plasma levels of amino acids and their derivates were observed in PWH with cirrhosis (**Figure 3A**). Within the lipidomic profile, phosphatidylcholines (PC) and phosphatidylethanolamines (PE) were the most prevalent lipid species identified, with chain length modification and desaturation being the most common alterations observed (**Figure 4A**).

Regarding chemical composition, most PC species were polyunsaturated (64.3%), followed by monounsaturated (21.4%) and saturated (14.3%). In contrast, all PE and phosphatidylinositol (PI) species, as well as all but one of phosphatidylserine (PS) species, were polyunsaturated (**Supplementary Data 7A**).

### Metabolic profiling associated with cirrhosis five years post-HCV therapy

OPLS-DA models adequately separated patients according to cirrhosis status, identifying 229 plasma metabolites with VIP scores >1 (**Figures 1B**, **2B**). Of these, 69 features were significantly associated with cirrhosis, with 20 found at lower levels (AMR<1), and 49 at higher levels (AMR≥1). The distribution of significant metabolites and lipids is shown in **Figure 3B**, with full association data provided in **Supplementary Data 8**.

At five years, individuals with cirrhosis exhibited lower plasma levels of amino acids and their derivatives (**Figure 3B**). Consistent with the one-year findings, PC and PE were the predominant lipid species associated with cirrhosis. The most frequent lipid modifications included chain length modifications, desaturation, and fatty acid (FA) additions (**Figure 4B**).

In terms of glycerophospholipids composition, a marked shift was observed in PC species compared to one-year time point: saturated PCs were no longer detected, while the proportions of polyunsaturated (73.3%) and monounsaturated (26.7%) species increased. No relevant compositional changes were found for PE, PS and PI (**Supplementary Data 7B**).

### Within-individual paired analysis of metabolic changes

When analyzing the temporal evolution of the significant metabolites identified at both time points, in relation to LSM (n=24 individuals with paired samples), several glycerophospholipids showed a significantly stronger association with LSM at year five than at year one (AMR >1). Conversely, the association between several amino acids and LSM was significantly attenuated over time (AMR <1) (**Supplementary Data 9**).

## Discussion

This study is, to our knowledge, the first to characterize the plasma metabolic profile in PWH with cirrhosis after long-term HCV cure. We found a persistent metabolic dysregulation up to five years post-HCV eradication, marked by elevated glycerophospholipids (PC, PE, PS and PI) and reduced amino acids and triglycerides. Over time, we also observed dynamic shifts in lysophospholipids (LPC, LPE and LPI), fatty acyls, and sphingolipids (SM and Cer). These disturbances suggest a lasting metabolic footprint beyond viral clearance. Consistent with post-injury metabolic reprogramming, these systemic glycolitic and lipid shifts likely reflect ongoing chronic immune activation and inflammatory environment that may continue to drive fibrogenesis despite SVR (23).

A key finding of our study is the altered plasma amino acid profile in PWH with cirrhosis, persisting up to five years post-HCV eradication. We observed elevated levels of hydroxyproline, citrulline, and arginine, together with reduced levels of essential and branched-chain amino acids (BCAAs) (e.g. L-valine, L-lysine, and L-tryptophan).

Notably, increased arginine and citrulline, were already evident one year after treatment, suggesting early and sustained metabolic dysregulation despite viral clearance. These patterns align with reports linking arginine and citrulline levels to urea cycle and nitric oxide metabolism dysfunction in advanced liver disease (24). Impaired mitochondrial function disrupts citrulline-to-arginine conversion in the urea cycle, leading to citrulline accumulation and arginine diversion toward nitric oxide. Nitric oxide is a crucial immunomodulator involved in vascular dysregulation, immune dysfunction, oxidative stress, and has been implicated in complications of advanced liver disease, including hepatic encephalopathy and portal hypertension (25, 26). Reduced plasma levels of BCAAs, which are essential for lymphocyte proliferation, maturation, and effector functions, may signal an impaired immune regenerative capacity (27). Also, BCAAs have consistently been associated with liver dysfunction and hepatic encephalopathy (25, 28, 29). As BCAAs regulate gene expression, protein metabolism, apoptosis and hepatocyte regeneration, their depletion may signal impaired hepatocyte regenerative capacity in patients with advanced chronic liver disease (30). Lower circulating BCAAs may also reflect increased skeletal muscle uptake and mitochondrial impairment in hepatocytes (31). Elevated plasma levels of amino acids, such as arginine and citrulline, are likely related to the cirrhotic state of the liver, which disrupts the urea cycle, impairs ammonia clearance and may lead to hyperammonemia. In contrast, the lower plasma levels of BCAAs observed at five years may reflect long-term metabolic dysfunction, as BCAA depletion compromises protein synthesis and promotes metabolic shift toward glutamine synthesis, a compensatory mechanism involved in ammonemia detoxification (32, 33). Together, these findings suggest that the different amino acid profiles observed at one and five years may reflect progressive disturbances in metabolic homeostasis despite HCV cure.

We found that PWH with cirrhosis showed sustained elevations in plasma levels of PC, PE, PS and PI, with little changes between one- and five-year post-treatment, while LPC, LPE and LPI levels remained largely unchanged. This suggest a stable pro-inflammatory glycerophospholipid profile associated with cirrhosis, consistent with prior links between LPC/LPE and hepatocyte lipotoxicity, pro-fibrogenic signaling, and sphingomyelin-ceramide axis alterations in fibrotic and cirrhotic states (34). Although lipidomic studies in liver cirrhosis are limited, our data align with reports connecting glycerophospholipid dysregulation to liver disease and malignancies (35–37). For example, Clària et al. observed reduced conversion of PC to LPC in acute-on-chronic liver failure (38), and Cotte et al. reported increased PC and decreased LPC levels in cirrhotic patients with HCC. Specific changes we observed, such as increased PC 16:0/16:1 and lower LPC 20:4, have also been described in cirrhosis (35), while elevated PI plasma levels has been associated with cirrhosis by Meikle et al. (39). Together, these findings support a persistent lipidomic signature in cirrhosis that may contribute to ongoing liver injury or systemic inflammation, highlighting potential targets for monitoring and therapy.

Although the overall glycerophospholipid profile remained stable, a key shift appeared five years post-therapy: increased desaturation of PC and PE species. Dysregulated desaturase activity is a hallmark of chronic liver disease, and altered phospholipid desaturation disrupts the balance between polyunsaturated and monounsaturated fatty acids in membrane phospholipids, a process known to affect lipid metabolism and contribute to aging and disease progression (40), promoting pro-inflammatory n-6 eicosanoid formation and increasing susceptibility to lipid peroxidation (34). This increased unsaturation of phospholipids sensitizes membranes to oxidative damage, a process well documented in advanced liver disease and decompensated cirrhosis (41). Oxidative lipid alterations have similarly been described across liver diseases, including decompensated cirrhosis (41–43). Thus, our findings suggest that these changes in glycerophospholipid composition are not markers of recovery but may reflect persistent metabolic disturbances that may drive liver disease progression long after HCV eradication.

A notable finding was the sustained reduction in TG species at both one and five years post-HCV therapy, undetected by routine biochemical tests that showed stable total plasma TG levels. This aligns with large-scale lipidomic studies reporting widespread TG species perturbations in cirrhosis (34). As the liver plays a central role in TG synthesis, secretion, and very-low-density lipoprotein handling (23, 44), our findings support prior evidence of lower plasma TG levels in cirrhosis (45). Clinically, reduced TG levels has been associated with worse prognosis in HCC and advanced liver diseases (34, 46), underscoring its value as a prognostic marker. Thus, persistent TG depletion may reflect latent metabolic imbalance and ongoing hepatic dysfunction, offering potential as a sensitive long-term biomarker in liver disease management.

We also found stage-dependent alterations in sphingolipid metabolism, particularly in plasma levels of SM and Cer. Sphingolipids are bioactive lipids regulating membrane structure, immune cell apoptosis, inflammation and hepatic function (47). Prior lipidomic studies show SM increases in early phases or after interventions, while progressive fibrosis and cirrhosis are marked by Cer reductions and broader alterations in sphingolipid disturbances (34). Consistent with this, we observed elevated SM levels at one year post-therapy, whereas these levels were not significantly altered in the five-year cross-sectional assessment. This aligns with previous reports describing SM variations after HCV treatment and in advanced disease (48). In parallel, the five-year evaluation showed a profile of lower Cer levels in PWH with cirrhosis, aligning with evidence that lower ceramides correlate with fibrosis severity and increased HCC risk (49). The pronounced decrease in Cer at five years suggests persistent inflammation and ongoing liver disease progression despite viral clearance.

Our data also revealed elevated levels of acylcarnitines in plasma in PWH with cirrhosis, evident only five years post-HCV clearance, suggesting persistent mitochondrial dysfunction. Acylcarnitines are key to fatty acids β-oxidation, transporting fatty acids chains into mitochondria for energy production (50, 51). Normally, tightly regulated elevated medium- and long-chain acylcarnitines are a hallmark of impaired β-oxidation and oxidative stress, reported in decompensated cirrhosis and acute hepatic failure (52–54). Their progressive accumulation in our study population likely reflects ongoing mitochondrial and hepatic dysfunction long after HCV eradication.

## Conclusions

In conclusion, PWH with cirrhosis after HCV cure show a persistently altered metabolic profile, with most alterations remaining stable for up to five years. Newly emerging metabolic disturbances at this stage suggest ongoing systemic inflammation and liver disease progression despite HCV clearance. Due to the limited information from long-term metabolomic studies in PWH following HCV eradication, our results are interpreted in the context of general liver cirrhosis. These findings underscore the need for long-term monitoring of this high-risk population, and support comprehensive metabolic profiling as a tool to identify individuals at risk of persistent immune dysfunction and to better understand the underlying pathophysiology of liver disease after HCV clearance.

## Strengths and limitations

The main limitations are the modest sample size, which may have reduced the statistical power and patient retention issues that limited paired sampling. In addition, the cross-sectional design at each time point prevents causal inference. Moreover, the absence of an HIV-negative control group with cirrhosis prevents us from definitively attributing the observed metabolic alterations specifically to the effect of HIV infection. Finally, the number of covariates adjusted for in our analyses was limited by the modest sample size. Consequently, important HIV-specific variables, such as specific ART regimens and CD4+ T cell counts, were not included in the models. Nevertheless, although residual confounding cannot be completely excluded, the potential confounding effect of these variables is likely limited, as they did not show significant differences between the cirrhotic and non-cirrhotic groups. Despite these limitations, the study has notable strengths. Most importantly, it provides the first long-term metabolomic follow-up of PWH with cirrhosis after HCV cure, offering insights into their evolving metabolic landscape. This design enabled us to characterize a dynamic metabolic signature that shorter-term or less targeted studies would likely miss.

## Data Availability

The raw data supporting the conclusions of this article will be made available by the authors upon reasonable request, without undue reservation.

## Appendix

### Members of the Marathon Study Group

**Hospital General Universitario Gregorio Marañón, Madrid:** A Carrero, P Miralles, JC López, F Parras, B Padilla, T Aldamiz-Echevarría, F Tejerina, C Díez, L Pérez-Latorre, C Fanciulli, I Gutiérrez, M Ramírez, S Carretero, JM Bellón, J Bermejo, and J Berenguer.

**Hospital Universitario La Paz, Madrid:** V Hontañón, JR Arribas, ML Montes, I Bernardino, JF Pascual, F Zamora, JM Peña, F Arnalich, M Díaz, J González-García.

**Hospital Universitari Vall d’Hebron, Barcelona:** E Van den Eynde, M Pérez, E Ribera, M Crespo.

**Hospital Universitario Príncipe de Asturias, Alcalá de Henares:** A Arranz, E Casas, J de Miguel, S Schroeder, J Sanz.

**Hospital Donostia, San Sebastián:** MJ Bustinduy, JA Iribarren, F Rodríguez-Arrondo, MA Von-Wichmann.

**Hospital Universitario de La Princesa, Madrid:** J Sanz, I Santos.

**Hospital Clínico San Carlos, Madrid:** J Vergas, MJ Téllez.

**Hospital Clínico Universitario, Valencia:** A Ferrer, MJ Galindo.

**Hospital Universitario Ramón y Cajal, Madrid:** JL Casado, F Dronda, A Moreno, MJ Pérez-Elías, MA Sanfrutos, S Moreno, C Quereda.

**Hospital General Universitario, Valencia:** L Ortiz, E Ortega.

**Fundación SEIMC-GESIDA, Madrid:** M Yllescas, P Crespo, E Aznar, H Esteban.
